# Comparison of depression care provided in general practice in Norway and the Netherlands: registry-based cohort study (The Norwegian GP-DEP study)

**DOI:** 10.1186/s12913-022-08793-7

**Published:** 2022-12-07

**Authors:** Anneli Borge Hansen, Valborg Baste, Øystein Hetlevik, Tone Smith-Sivertsen, Inger Haukenes, Derek de Beurs, Mark Nielen, Sabine Ruths

**Affiliations:** 1 Research Unit for General Practice, NORCE Norwegian Research Centre, Bergen, Norway; 2 Department of Global Public Health and Primary Care, University of Bergen, Bergen, Norway; 3 Division of Psychiatry, Haukeland University Hospital, Bergen, Norway; 4 Department of Mental Health and Prevention, Trimbos Institute, Utrecht, The Netherlands; 5 Netherlands Institute for Health Services Research, Utrecht, The Netherlands

**Keywords:** Mental health, Large database research, Health services research, Depression, General practice, Antidepressive agents, Drug therapy, Prescriptions

## Abstract

**Background:**

Depression is highly prevalent in general practice, and organisation of primary health care probably affects the provision of depression care. General practitioners (GPs) in Norway and the Netherlands fulfil comparable roles. However, primary care teams with a mental health nurse (MHN) supplementing the GP have been established in the Netherlands, but not yet in Norway. In order to explore how the organisation of primary mental care affects care delivery, we aimed to examine the provision of GP depression care across the two countries.

**Methods:**

Registry-based cohort study comprising new depression episodes in patients aged ≥ 18 years, 2011–2015. The Norwegian sample was drawn from the entire population (national health registries); 297,409 episodes. A representative Dutch sample (Nivel Primary Care Database) was included; 27,362 episodes. Outcomes were follow-up consultation(s) with GP, with GP and/or MHN, and antidepressant prescriptions during 12 months from the start of the depression episode. Differences between countries were estimated using negative binomial and Cox regression models, adjusted for patient gender, age and comorbidity.

**Results:**

Patients in the Netherlands compared to Norway were less likely to receive GP follow-up consultations, IRR (incidence rate ratio) = 0.73 (95% confidence interval (CI) 0.71–0.74). Differences were greatest among patients aged 18–39 years (adj IRR = 0.64, 0.63–0.66) and 40–59 years (adj IRR = 0.71, 0.69–0.73). When comparing follow-up consultations in GP *practices*, including MHN consultations in the Netherlands, no cross-national differences were found (IRR = 1.00, 0.98–1.01). But in age-stratified analyses, Dutch patients 60 years and older were more likely to be followed up than their Norwegian counterparts (adj IRR = 1.21, 1.16–1.26). Patients in the Netherlands compared to Norway were more likely to receive antidepressant drugs, adj HR (hazard ratio) = 1.32 (1.30–1.34).

**Conclusions:**

The observed differences indicate that the organisation of primary mental health care affects the provision of follow-up consultations in Norway and the Netherlands. Clinical studies are needed to explore the impact of team-based care and GP-based care on the quality of depression care and patient outcomes.

## Background

Globally, depressive disorders account for the largest proportion of disability-adjusted life years (DALYs) related to mental disorders, i.e., 37% in 2019 [1]. General practitioners (GPs) play a key role in providing treatment and follow-up to patients with depression, usually consisting of psychological therapy and/or antidepressant drug therapy. Most antidepressants are prescribed by GPs [2, 3], but the prescribing levels vary considerably between countries [4–8]. Recent studies indicate trends towards less drug treatment in the Netherlands, UK, and Norway [7, 9, 10], while treatment rates remained stable in the US [8]. The number of GP contacts for depression increased in the Netherlands [4, 9] but was unchanged in Norway [10].

Although many European countries have a strong primary care sector, the primary health care services are organized differently. For instance, primary care teams (PCTs) with registered nurses and other professionals who complement the GP have been established in the Netherlands, Canada, and Australia [4, 11–16]. It is reasonable to expect that the organisation of primary care services affects the provision of depression care in general practice, however, knowledge is scarce regarding depression care. Comparison of care delivery in two countries offering public primary health care services but with different organisation of primary mental care may generate new knowledge to guide organisational changes and improving the management of a large group of patients.

Norway and the Netherlands offer universal health care, ensuring all residents equal access to low-cost medical care [17]. GP care in Norway is covered by the National Insurance Scheme. Patients older than 15 years must pay an out-of-pocket fee for consultations, up to an annual maximum sum (219 € in 2014). In the Netherlands, GP care is covered by private mandatory insurance with no deductible for GP visits. GPs in both countries fulfil comparable roles, having fixed, personalised patient lists, and acting as gatekeepers to secondary care. PCTs have been established in the Netherlands, but not yet in Norway. Since 2008, mental health nurses (MHNs) have been gradually introduced in Dutch general practices as part of health policy aiming to improve early identification and treatment of mental health problems in primary care [18]. Since 2014, a GP working in an average size practice can be supported by an MHN for approximately one day a week [4]; more than 80% of GP practices have an MHN affiliated [19]. Dutch MHNs have received higher education in nursing or psychology, and their main tasks are to perform diagnostic assessment, and to deliver short-term counselling or psychoeducation to patients with psychological symptoms or social problems. MHNs work under the supervision of the GP. In general, the GP decides after a first consultation if a patient should visit the MHN. GPs can also decide to treat patients themselves or refer patients to specialised mental healthcare. MHN do not prescribe medication.

In order to explore how different organisation of primary mental care affects care delivery, we aimed to examine the provision of depression care to patients with a new depression episode in general practice in Norway and the Netherlands, 2011–2015.

## Methods

### Design

We conducted a cohort study based on registry-data from primary care in Norway and the Netherlands, for the period 2011–2015. We compared the provision of GP depression care for 12 months from the date of the depression diagnosis (index date) between the countries.

### Data sources

In Norway, information from national registries was linked at the individual patient level, using the (encrypted) unique personal identification number assigned to all residents. The Norwegian study population was drawn from the *Population Registry*. We obtained information regarding gender, year of birth, death, and emigration for all residents aged 18 years and older. The *Control and reimbursement of health care claims (KUHR)* database stores data on all fee-for-service claims from public primary care providers. For each consultation with a GP, we obtained information on the date consultation and all diagnoses according to the International Classification of Primary Care (ICPC), as recorded by the GPs. The *Norwegian Patient Registry (NPR)* comprises information on all patient contacts with refundable secondary health care, for administrative and funding purpose. We obtained information on date of depression contact with secondary mental health care providers, with diagnoses according to the International Classification of Disease (ICD). The *Norwegian prescription database (NorPD)* stores information on all prescription drugs dispensed to patients treated in ambulatory care [20]. For each prescription of an antidepressant drug, NorPD provided information on date of dispensing and generic drug information (Anatomical Therapeutic Chemical (ATC) code). Data sources and variables have been described previously [10].

The Dutch study population was drawn from the *Primary Care Database at Netherlands Institute for Health Services Research (PCD-Nivel)* [21]. PCD-Nivel stores routinely recorded data from health care providers’ electronic health records. The database includes a voluntary, representative sample of approximately ten percent of all Dutch GP practices fulfilling the following criteria: ≥ 500 list patients, morbidity registration during ≥ 46 weeks per year, and ≥ 70% encounters recorded with an ICPC code. The patient population of Nivel Primary Care Database (Nivel-PCD) is representative of the Dutch population regarding sex and age. There is some overweight of group practices in the Nivel PCD, while practices in urban locations are underrepresented [9]. GPs structure their electronic health records around *episodes of care* that contain all patient encounters, prescribed medication, and interventions related to the same health problem [22]. For all recorded depression episodes, we obtained information on consultations with GP and MHN, all diagnoses recorded according to the ICPC system, and prescriptions of antidepressant drugs. All practices in PCD-Nivel were included in this study, both practices with and without a mental health nurse. A study by Magnee et al. based on Nivel-PCD data in the same study period as the present study showed that in 2011, 41% of general practices employed a MHN and 88% in 2015 [9]. In the present study, no information was available regarding whether a GP practice had a MHN affiliated.

All data was stored and analysed at a safe server at the University of Bergen.

### Study population

The source population at risk in Norway comprised the entire population aged 18 years and older. First, we identified all individuals with one or more depression diagnoses recorded in general practice (GP consultation with the ICPC code Depression in KUHR) during 2011–2015. Second, to establish a cohort of patients with a *new* depression episode, washout was performed for patients with a depression diagnosis in general practice (any encounters registered with ICPC code Depression in KUHR) and/or secondary care (ICD 10 codes F32, F33, F34 or F41.2 in NPR), and/or dispensed antidepressant drug treatment for depression (ATC code N06A in NorPD) during 12-months *prior to index date* [10]*.* The 12-month contact-free interval was defined according to an algorithm published by Nielen and coworkers [22]. We thus identified 256,956 unique patients with new depression episodes. Out of these, 32,187 incurred two or more depression episodes that were at least 12 months apart.

The Dutch study population was drawn from the PCB-Nivel, including data from 462 GP practices. First, we identified all patients aged 18 years and older recorded with one or more depression episodes during 2011–2015 that were at least 12 months apart. Second, to establish depression episodes starting with a GP consultation for depression (i.e., index contact), washout was conducted for depression episodes including index contacts with other health care providers than GP, or ICPC codes for diagnoses other than P76 Depression. We thus identified 25,804 unique Dutch patients with a new depression episode. Out of these, 1,508 incurred two or more depression episodes.

### Independent variables

Country (Norway and the Netherlands) was the independent variable. Patients’ gender (women, men), age, and comorbidity were covariates. We categorized age as 18–29, 30–39, 40–49, 50–59, 60–69, and ≥ 70 years. In the regression models, age was categorized as 18–39, 40–59, and ≥ 60 years. Based on patients’ ICPC codes recorded during 12 months from index date, comorbidity was identified according to an established list of 40 common, chronic conditions [23], and categorised as none, 1–2, and 3 + conditions.

### Outcome

We analysed follow-up consultations in general practice with and without including MHN information from the Netherlands, and treatment with antidepressants. Number of follow-up consultations with GP in both countries, and with MHN in the Netherlands, linked to ICPC code P76 Depression, during one year after the index date was counted. The variables were binary (yes/no) and count variable. Information on antidepressant drugs prescribed (Netherlands) or dispensed (Norway) during 12-months from index date (yes/no) and time to first prescription (immediate: within one week after first consultation, or non-immediate: at least one week after first consultation) was used. The drugs were categorised according to the ATC classification system as antidepressants (ATC code N06A). Number of days from index date to first drug prescribing/dispensing was categorised as 0–7, 8–31, 32–183, and 184–365 days.

### Statistical analysis

Descriptive statistics was used to examine the distribution of age, gender, and comorbidity given by numbers and percentages of patients, by country.

By country, GP and MHN (Netherlands) follow-up consultation (yes/no) and antidepressant medication during 12 months from index date, were provided by percentages of episodes and mean numbers of consultations, for age-groups, gender, and comorbid conditions.

Antidepressant medication, and number of days from index date to first drug prescribing/dispensing during 12 months from index date in (categories), were provided by numbers and percentages of depression episodes, by country. Differences between countries in prescription of antidepressants (immediate prescription, non-immediate prescription, no prescription) in depression episodes with or without follow-up consultations were analysed with chi-square test.

Negative binomial regression was performed to explore the likelihood of having follow-up consultation(s) with GP, and with GP and/or MHN (count variables) in the Netherlands versus Norway (reference). Results are presented as incidence rate ratios (IRRs) with 95% confidence intervals (CIs), crude and adjusted for gender, age, and comorbidity. To account for recurrent episodes of depression in some patients, cluster function in STATA was used. Cox regression was used to estimate the likelihood of receiving antidepressant drugs, taking into account time (days) from index date to first prescription. Results are presented as hazard ratios (HRs) crude and adjusted for gender, age, and comorbidity, with 95% CIs. Further, negative binomial regression and Cox regression were performed for three age strata, crude and adjusted for gender and comorbidity.

For all statistical analyses, α = 0.05 was used as significance level. The data were analysed using STATA/SE version 16.1 (Stata Statistical Software).

## Results

The Norwegian study population comprised 291,713 depression episodes in 256,956 unique individuals, 63.0% were women (Table 1). The Dutch study population comprised 27,362 episodes in 25,804 unique individuals, 62.6% were women. Dutch patients were older (*p* ≤ 0.001) and had more comorbidities (*p* ≤ 0.001) compared to Norwegian patients. Number of comorbid conditions increased with increasing age in Norway and the Netherlands (*p* ≤ 0.001, not tabulated). In both countries, patients with more than one depression episode during the study period had more comorbid conditions compared to their respective study populations (68.4% vs 53.4% in the Netherlands, and 65.7% vs 49.1% in Norway), not tabulated.

**Table 1:** Characteristics of patients with one or more new depression episodes^a^ in general practice in Norway and the Netherlands, 2011–2015

**Number of patients**	**Norway**^**b**^ *N* = 256,926		**The Netherlands**^**c**^ *N* = 25,804	
	**n**	**%**	**n**	**%**
**Age group, years**				
18–29	60,400	23.5	4,020	15.6
30–39	52,108	20.3	4,327	16.8
40–49	54,981	21.4	5,775	22.4
50–59	42,889	16.7	5,649	21.8
60–69	25,371	9.9	3,188	12.4
70 +	21,177	8.2	2,845	11.0
**Gender**				
Women	161,952	63.0	16,142	62.6
Men	94,974	37.0	9,662	37.4
**Comorbid conditions**				
None	125,337	48.8	11,803	45.7
1–2	116,057	45.2	11,594	44.9
3 +	15,532	6.0	2,407	9.4

^a^Twelve-month washout for depression diagnosis in general practice and/or secondary care and/or antidepressant drug prescription prior to index date

^b^Source population: entire population; 32,187 patients had more than one episode

^c^Representative sample of residents (Primary Care Database at Netherlands Institute for Health Services Research); 1,508 patients had more than one episode

Table 2 shows that 65.4% of new depression episodes among Norwegian patients were followed up with GP consultation(s). Among Dutch patients, 44.8% of the episodes were followed up by GP only, 4.2% by MHN only, and 12.5% by both GP and MHN. In Norway, the percentage of episodes followed up with GP consultation(s) decreased with increasing patient age, except for those aged 18–29. In the Netherlands, follow-up consultations with GP increased, while follow-up consultations with MHN only, and with both GP and MHN decreased with increasing patient age. Slightly more female than male patients in Norway had GP follow-up consultations. Among patients with three or more comorbid conditions the percentage of having follow-up consultation(s) with GP was slightly lower in Norway and slightly higher in the Netherlands, compared to patients with no comorbidity.

**Table 2:** GP and MHN (Netherlands) consultations and prescriptions, during 12 months from index date, for new depression episodes^a^ in general practice, percentages and mean given for age groups, gender and comorbid conditions, by country

	**Norway: 291,713 depression episodes**			**The Netherlands: 27,362 depression episodes**					
	**Follow-up consultation**			**Follow-up consultation**					
	**Episodes**	**GP**	**Antidepressant**	**Episodes**	**GP only**	**MHN only**	**GP and MHN**	**GP and/or MHN**	**Antidepressant**
	**n**	**(n) %**	**%**	**n**	**(n) %**	**(n) %**	**(n) %**	**(n) %**	**%**
		(190,850) 65.4	47.4		(12,261) 44.8	(1,156) 4.2	(3,425) 12.5	(16,285) 61.5	59.5
**Age, years**									
18–29	67,008	64.0	38.4	4,188	40.7	6.2	13.9	60.8	45.2
30–39	59,235	67.8	41.1	4,556	44.5	5.1	13.0	62.5	55.2
40–49	62,935	67.6	45.8	6,144	44.6	4.3	13.3	62.2	60.5
50–59	49,301	66.3	49.5	6,018	45.2	3.8	13.2	62.2	62.1
60–69	29,288	62.6	59.3	3,404	46.0	3.1	10.9	60.0	67.3
70 +	23,946	60.0	73.5	3,052	49.3	2.3	8.9	60.5	70.0
**Gender**									
Women	184,639	65.8	48.3	17,156	44.8	4.1	12.6	61.5	60.4
Men	107,074	64.7	46.0	10,206	44.8	4.4	12.5	61.6	58.0
**Comorbid conditions**									
None	137,203	65.7	41.1	12,294	43.5	5.1	13.0	61.6	54.7
1–2	134,384	66.5	51.6	12,378	45.8	3.7	12.3	61.8	62.6
3 +	20,126	63.0	62.6	2,690	46.5	2.6	11.0	60.1	67.4

*GP* General practitioner, *MHN* Mental health nurse

^a^Twelve-month washout for depression diagnosis in general practice and/or secondary care and/or antidepressant drug prescription prior to index date

In both countries, the percentage of depression episodes treated with antidepressant drugs increased with increasing patient age and increasing numbers of comorbid conditions. Antidepressant medication was more commonly prescribed to women than to men in both countries.

The countries differed regarding treatment rates. Norwegian patients had more commonly follow-up consultation with the GP (65.4% vs. 57.3% (i.e., 44.8% + 12.5%) of episodes), Table 3. The adjusted incidence rate ratio of treatment in the Netherlands compared to Norway was IRR = 0.73 (95% CI 0.72–0.74), Table 4. Age stratified analysis showed that all age groups in the Netherlands were less likely to receive GP follow-up consultation(s), compared to Norway. However, the differences were most prominent among patients aged 18–39 years (IRR = 0.64, CI 0.63–0.66) and 40–59 years (IRR = 0.71, CI 0.69–0.73). Comparing follow-up consultations in GP *practices*, including MHN consultations in the Netherlands, no cross-national differences were found (IRR = 1.00, 0.98–1.01). Age-stratified analysis showed that patients aged 18–39 years had a lower likelihood of receiving GP/MHN follow-up consultations in the Netherlands compared to Norway (IRR = 0.92, CI 0.89–0.94), while patients 60 years and older had a markedly higher likelihood of receiving GP/MHN follow-up consultations (IRR = 1.21, 1.16–1.26), in the Netherlands compared to Norway.

**Table 3:** Consultations and prescriptions during 12 months from index date, for new episodes of depression^a^ in general practice, by country

	**Number of new depression episodes, 2011–2015**					
	**Norway** *N* = 291,713^b^			**The Netherlands** *N* = 27,362^b^		
	**n**	**%**	**mean**	**n**	**%**	**mean**
**Consultation**						
Index consultation only	100,863	34.6		10,520	38.4	
Follow-up consultation						
GP only	190,850	65.4	2.4	12,261	44.8	1.32
MHN only				1156	4.2	0.13
GP and MHN				3425	12.5	0.96
**Antidepressant medication**	138,395	47.4		16,285	59.5	
Number of days from index date to first prescription, among patients treated with drugs						
0–7	65,979	47.7		8,584	52.7	
8–31	22,163	16.0		2,891	17.8	
32–183	38,355	27.7		3,761	23.1	
184–365	11,898	8.6		1,049	6.4	

^a^Twelve-month washout for depression diagnosis in general practice and/or secondary care and/or antidepressant drug prescription prior to index date

^b^Number of patients with one or more episodes of depression: 256,956 in Norway; 25,804 in the Netherlands

**Table 4:** Likelihood^a^ of having follow-up consultation with GP, with GP and/or MHN, and of receiving antidepressant drug, during 12 months from index date, for a new depression episode^b^ in the Netherlands compared to Norway

	**Follow-up consultation with GP**^**c**^				**Follow-up consultation with GP and/or MHN**^**d**^				**Antidepressant drug**			
Netherlands (Norway = reference)	**Crude IRR**	**95% CI**	**Adj IRR**^**e**^	**95% CI**	**Crude IRR**	**95% CI**	**Adj IRR**^**e**^	**95% CI**	**Crude HR**	**95% CI**	**Adj HR**^**d**^	**95% CI**
All patients	0.72	0.71–0.74	0.73	0.71–0.74	0.99	0.97–1.01	1.00	0.98–1.01	1.41	1.39–1.43	1.32	1.30–1.34
Stratified for age group												
18–39	0.64	0.62–0.66	0.64	0.63–0.66	0.92	0.89–0.94	0.92	0.89–0.94	1.37	1.33–1.42	1.39	1.34–1.43
40–59	0.71	0.69–0.73	0.71	0.69–0.73	0.98	0.95–1.01	0.98	0.95–1.01	1.50	1.46–1.53	1.50	1.46–1.53
60 +	0.95	0.91–0.98	0.95	0.92–0.99	1.20	1.15–1.25	1.21	1.16–1.26	1.11	1.07–1.14	1.10	1.07–1.14

*GP* General practitioner, *MHN* Mental health nurse

^a^Results from negative binomial regression estimating incidence rate ratios (IRRs) with 95% confidence interval (CI). Results from Cox regression estimating hazard ratios (HRs) with 95% CI

^b^Twelve-month washout for depression diagnosis in general practice and/or secondary care and/or antidepressant drug prescription prior to index date

^c^Follow-up consultation with GP, regardless of whether the patient also received follow-up with MHN

^d^Follow-up consultation with GP only, MHN only, or GP and MHN

^e^Estimates for all patients are adjusted for gender, age, and comorbidity; estimates for age strata are adjusted for gender and comorbidity

**Fig. 1:**
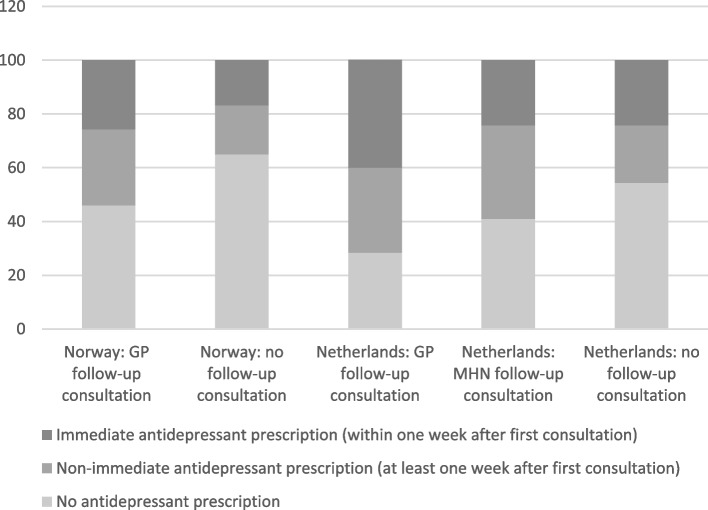
Prescription of antidepressants in depression episodes with or without follow-up consultations (not adjusted for year of episode). GP: general practitioner, MHN: mental health nurse. Legend: GP: general practitioner, MHN: mental health nurse

Antidepressant drug treatment was more commonly provided to Dutch than to Norwegian patients (59.5% vs. 47.4% of the episodes, *p* ≤ 0.001), and prescribed/dispensed earlier after index date to Dutch than Norwegian patients (*p* ≤ 0.001), Table 3. Figure 1 shows that antidepressants were prescribed more commonly to Dutch patients than to Norwegian patients; this applied both to patients with and without follow-up consultations. Dutch patients who only consulted their GP received antidepressants more commonly and earlier compared to those (also) consulting a MHN. These differences were statistically significant, *p* ≤ 0.001.

The adjusted HR was 1.32 (1.30–1.34) for receiving drug treatment in Netherland compared to Norway, Table 4. Age stratified analysis showed that all age groups had a higher HR for receiving drug treatment in the Netherlands, compared to Norway. However, the difference was most prominent in patients aged 18–59 years.

## Discussion

### Summary

In a cohort of adult patients with new depression episodes, Dutch patients were less likely to receive follow-up consultation with GP compared to patients in Norway; differences were greatest among those aged 18–59 years. No cross-national differences were found when comparing follow-up consultation(s) in GP *practices*, including MHN consultation(s) in the Netherlands; however, Dutch patients 60 years and older were more likely to receive follow-up. Patients in the Netherlands compared to Norway were more likely to receive antidepressant drugs.

### Strengths and limitations

Combining nationwide registry data from the primary care services in Norway and a large, representative sample from the Nivel Primary Care Database in the Netherlands provides a unique source of information, eliminating recall bias. The primary care databases used in this study differ in extent, but they are representative of the populations in Norway and the Netherlands, respectively.

Information on GP-diagnosed depression is another strength. A new depression diagnosis was defined as a GP-consultation with the ICPC code P76, with 1-year washout. The diagnostic criteria for depression among adults and recommendations in the guidelines for primary mental health care in Norway [24] and the Netherlands [25] are similar. However, it is possible that adherence to guidelines among GPs varies between the two countries. Further, we cannot know for sure whether GPs in Norway carry out coding of depression diagnoses differently than GPs in the Netherlands, or whether GPs in the Netherlands who supply data to Nivel-PCD carry out diagnostic coding differently than GPs not participating in Nivel-PCD. Differing diagnostic and coding behavior may, therefore, challenge the internal validity. However, potential misclassification by the GP would be non-differential and probably distributed randomly across the GP practice populations in both countries. Information on the severity of depression was lacking, as the ICPC-system does not allow for such grading. This is a limitation because severity influences GPs’ treatment decisions. However, there is no reason to assume that severity differs between the Dutch and the Norwegian study populations. Provided representativity of the two study samples, and the same case definition in both countries, we consider the study populations comparable but not identical.

The NorPD in Norway contains data on antidepressant drugs *dispensed* while the Nivel-PCD in the Netherlands contains data on antidepressant drugs *prescribed*, which challenges the comparison of treatment levels across the countries. Although low out-of-pocket payment makes medication for depression easily available in both countries, we cannot estimate the prevalence of primary non-compliance, i.e., patients not collecting their prescribed drugs. No information was available on the appropriateness of drug treatment provided. Guidelines for prescription of antidepressant agents to patients with depression are similar in Norway and the Netherlands. However, we cannot rule out that adherence to guidelines among GPs differs between the two countries.

Differences in referral rates could be a possible explanation for (some of the) the differences in antidepressant drug treatment between Norway and the Netherlands. However, since information on referrals to secondary mental health care was not included in the dataset from Nivel-PCD, we could not explore this further.

The results of this study could be transferable to countries where GPs fulfil comparable roles to the Norwegian and Dutch GPs (having fixed, personalized patient lists, and acting as gatekeepers), such as, e.g., Sweden, the UK and Canada. The results are less comparable with countries with different organisation of primary health care services, or direct access to specialised mental health care.

### Interpretation of findings and comparison with existing literature

#### Study population

The gender distribution in the study populations is consistent with previous research [1, 5, 26], reflecting a higher prevalence of depression [1] and more doctor-seeking [27] among women compared to men. Although depression is more common among older people according to prevalence studies of whole populations [28, 29], patients aged 60 + constitute a relatively small proportion of the study population. This may reflect a more prolonged or chronic course of depression among older people, with relatively fewer new episodes. Further, less doctor-seeking for mental health problems among older people has been documented, as they preferred self-management strategies that aligned with their lived experiences and self-image [30].

#### Follow-up consultations

Overall, patients in the Netherlands compared to Norway were less likely to receive follow-up consultation(s) with the GP, but no cross-national differences were found when comparing follow-up consultation(s) in GP *practices*, including MHN consultation(s) in the Netherlands. These findings support that MHNs significantly supplement Dutch GPs in following up patients with depression. It has been documented that the total number of GP contacts for depressive disorder, feeling depressed, and other mental health conditions increased after implementing a collaborative depression programme and integrating MHNs in general practices in the Netherlands [4, 12]. This supports that MHNs provide additional support and increased accessibility to mental health care in general practice, but not necessarily decrease the GP’s overall workload [4]. Furthermore, increased number of GP contacts for mental health-related conditions reflects the objectives of the Dutch government and health insurance companies to delegate mental health care from secondary care to primary care [12]. In Norway, where PCTs have not yet been established, numbers of GP contacts for new depression episodes remained unchanged in 2009–2015 [10].

Age-stratified analysis showed that depressed patients in the Netherlands compared to Norway were less likely to receive follow-up consultations with their GP, especially those younger than 60 years. Comparing follow-up consultations in GP *practices*, Dutch patients aged 18–39 years were less likely, and patients 60 years and older were markedly more likely to receive follow-up consultations with GP and/or MHN. Adjusting for comorbidity (registered in all GP contacts during 12 months from index date) did not alter the estimates; this was not surprising, as number of comorbid conditions increased with age. The findings may indicate that Dutch GPs entrust their younger and healthier patients with depression to MHNs, and follow-up their older and sicker patients themselves. This supports that follow-up with MHN is mostly relevant for younger or healthier patients [4]. In Norway, older patients, and those with multimorbidity received the least follow-up consultations with GP. There are no PCTs established in general practice in Norway yet, and older patients are less likely to be referred to secondary mental health care compared to working-aged patients [10]. This may imply that the oldest and sickest patients are less prioritized for the treatment of depression than younger and more healthy patients.

#### Antidepressant drug treatment

The antidepressant treatment rates found in this study are in line with the 50–60% rates reported in studies from general practice in Sweden and the UK [5, 7]. Approximately half of the study population received medication within one week after index date, in accordance with a Swedish registry-based study [5]. Magnee et al. found that introduction of MHN did not lead to a reduction in antidepressant prescriptions by the GP, but rather a postpone in prescription of antidepressants. In the current study we found that Dutch patients with GP consultations only received antidepressants more commonly and earlier compared to patients (also) consulting a MHN. Possible explanations for this difference may result from the use of antidepressant prescription for both anxiety and depressive disorders in Magnee’s study, compared to the use of antidepressant drugs for depression only in our study [9].

Age-stratified analysis showed that depressed patients in the Netherlands compared to Norway were more likely to receive antidepressant drugs, especially those younger than 60 years, and to start on drug treatment early. There may be cross-national differences contributing to the observed discrepancies, e.g., guidelines, doctors 'and patients' attitudes, and availability of non-pharmacological treatment modalities like cognitive techniques in primary and secondary health care. However, we cannot rule out that these discrepancies (in part) can be explained by different data sets, i.e., prescribed medication in the Netherlands versus collected medication in Norway. A systematic review of nonfulfillment of prescription medications from GPs and emergency care doctors in the US, Canada and European countries revealed that 11%-19% (median 15%) of prescriptions were actually not dispensed to the patients [31]. Even though none of the studies included in the review specifically addressed antidepressants, the cross-national difference found in this study is in the same range.

In both countries, older patients, and those with multimorbidity were more commonly treated with antidepressant drugs than younger or healthier patients, in line with a study from the UK [32]. We don’t know if the oldest or sickest patients had generally more severe or long-standing depression than other patient groups, which could explain the different treatment rates. The marked age gradient is of great concern because age-related changes and use of several drugs concomitantly entails an increased risk of adverse side effects [33]. Further, it has been documented that older or sicker patients with a new depression episode in Norway received more commonly pharmacological therapy and less commonly talking therapy or referral to secondary mental health care [10], even though patients of all ages prefer psychological approaches to medication [34]. This raises the question whether the observed discrepancies may reflect age discrimination (“ageism”) in two European countries with strong healthcare systems.

## Conclusion

The observed differences indicate that the organisation of primary mental health care affects the provision of follow-up consultations in Norway and the Netherlands. Clinical studies in countries with similar organization of primary care are needed to explore the impact of PCTs on the quality of depression care and patient outcomes. GPs should be aware that older patients and those with multimorbidity seem to receive less follow-up consultations, and more drug treatment for depression than other patient groups.

## Data Availability

The Norwegian datasets analyzed in this study are not publicly available due to restrictions only to be used under license for researchers in the current study, but are available from the corresponding author upon reasonable request and with included permission from Regional Ethical Committee for Medical and Health Research Ethics, Region West, Norwegian Data Protection Authority, Statistics Norway, the Norwegian Directorate of Health, and the Norwegian Institute of Public Health. The Dutch data sets analyzed in this study are not publicly available due to restrictions only to be used under license for researchers in the current study but are available from the corresponding author upon reasonable request and with included permission from Nivel Primary Care Database.

## Abbreviations

ATC: Anatomical Therapeutic Chemical
CI: Confidence Interval
DALY: Disability-adjusted life years
GP: General practitioner
HR: Hazard ratio
ICD: International Classification of Diseases
ICPC: International Classification of Primary Care
IRR: Incident rate ratio
KUHR: Control and Reimbursement of Primary Health Care Claims Database
MHN: Mental health nurse
NorPD: Norwegian Prescription Database
NPR: Norwegian Patient Registry
Nivel: Netherlands Institute for Health Services Research
PCD: Primary Care Database
PCT: Primary care team
SD: Standard Deviation
WHO: World Health Organisation
